# Efficacy and Safety of Nemolizumab in Patients With Prurigo Nodularis: A Systematic Review and Meta-Analysis of Randomized Controlled Trials

**DOI:** 10.7759/cureus.78761

**Published:** 2025-02-09

**Authors:** Kunal Sinha, Tanya Sinha, Neeharika Muppa, Nabeela Kanwal, Keron Blair, Sandipkumar S Chaudhari, Calvin R Wei, Adil Amin

**Affiliations:** 1 Dermatology, Redefine Skin Clinic, Patna, IND; 2 Internal Medicine, Tribhuvan University, Kathmandu, NPL; 3 Department of Medicine, St. George’s University School of Medicine, St. George’s, GRD; 4 College of Physicians and Surgeons Pakistan (CPSP), Pakistan Institute of Medical Sciences (PIMS) Hospital, Islamabad, PAK; 5 Medicine, American International School of Medicine, Georgetown, GUY; 6 Cardiothoracic Surgery, University of Alabama at Birmingham, Birmingham, USA; 7 Family Medicine, University of North Dakota School of Medicine and Health Sciences, Fargo, USA; 8 Research and Development, Shing Huei Group, Taipei, TWN; 9 Cardiology, Pakistan Navy Station (PNS) Shifa, Karachi, PAK

**Keywords:** il-31, itching, nemolizumab, prurigo nodularis, systematic review and meta analysis

## Abstract

This meta-analysis evaluated the efficacy and safety of nemolizumab in treating prurigo nodularis through a systematic review of randomized controlled trials. A comprehensive literature search was conducted across multiple databases, including PubMed, Embase, Cochrane Library, and Web of Science, identifying relevant studies until January 5th 2025. Four randomized controlled trials involving 859 participants were included in the final analysis. The primary outcomes assessed were itching response measured by the Worst Itch Numeric Rating Scale (WI-NRS) and Investigator's Global Assessment (IGA) success. Nemolizumab demonstrated significant improvement in itch response compared to control, with a risk ratio of 3.52 (95% CI: 2.48 to 5.02, p < 0.00001) and low heterogeneity (I² = 28%). Similarly, IGA success rates were notably higher in the nemolizumab group, with a risk ratio of 4.40 (95% CI: 2.86 to 6.75, p < 0.00001) and low heterogeneity (I² = 11%). While adverse events were slightly more frequent in the nemolizumab group, the difference was not statistically significant (RR: 1.11, 95% CI: 0.99 to 1.24). The analysis was limited by the small number of included trials, relatively short follow-up periods, and lack of subgroup analysis. Despite these limitations, the findings suggest that nemolizumab is an effective and well-tolerated treatment for prurigo nodularis. Further research with longer follow-up periods and larger, more diverse patient populations is recommended to establish the long-term efficacy and safety profile of nemolizumab in treating this condition.

## Introduction and background

Prurigo nodularis (PN) is a debilitating chronic skin disease characterized by intensely pruritic, hyperkeratotic nodules that predominantly affect the extremities [1]. The condition arises from a vicious itch-scratch cycle, where persistent pruritus triggers repeated scratching, leading to further skin damage and inflammation [2]. PN significantly impacts patients’ quality of life, contributing to sleep disturbances, emotional distress, and social isolation [3]. Despite its profound physical and psychological burden, the pathogenesis of PN remains incompletely understood. It is widely accepted that PN involves a complex interplay between immune dysregulation, neuroinflammation, and alterations in the skin barrier [4]. 

Current therapeutic options for PN are largely inadequate, with treatments often providing incomplete relief [5]. Conventional management strategies include topical corticosteroids, systemic immunosuppressants, and phototherapy, which primarily address inflammation rather than the underlying neuroimmune drivers of pruritus [6]. Advances in understanding the disease mechanisms have highlighted the critical role of interleukin-31 (IL-31) in mediating pruritic responses. IL-31, produced predominantly by activated Th2 cells, binds to its receptor expressed on sensory neurons and keratinocytes, perpetuating pruritus and inflammation [7]. This discovery has spurred interest in targeted biologic therapies that disrupt this pathway. 

Nemolizumab, a humanized monoclonal antibody targeting the IL-31 receptor A, represents a novel approach to managing PN by addressing both the itch and inflammatory components of the disease [8]. Clinical trials have demonstrated its potential to reduce pruritus severity, improve skin lesions, and enhance patients’ quality of life [9,10]. However, while individual studies suggest efficacy, the variability in trial designs, populations, and reported outcomes warrants a comprehensive evaluation of its overall therapeutic benefits and safety profile. 

This meta-analysis aims to systematically synthesize available evidence on the efficacy and safety of nemolizumab in patients with prurigo nodularis. By aggregating data from clinical trials, we seek to provide robust conclusions regarding its impact on pruritus, lesion improvement, and adverse events. This work not only addresses an important gap in the literature but also has the potential to inform clinical decision-making and guide future research in the field of pruritic dermatoses.

## Review

Methodology 

This systematic review and meta-analysis were performed and reported as per the guidelines of Preferred Reporting of Systematic Review and Meta-Analysis (PRISMA). 

Literature Search 

To find pertinent studies assessing the effectiveness and safety of nemolizumab in patients with prurigo nodularis, we carried out a thorough literature search. Several electronic databases, including PubMed, Embase, the Cochrane Library, and Web of Science, were searched. There were no limitations on language or publication status, and articles published between the databases' launch and January 5th were taken into consideration. In addition to Medical Subject Headings (MeSH) terms, the following keywords were utilised to find relevant articles: "nemolizumab," "prurigo nodularis," "chronic pruritus," "IL-31 receptor," "IL-31 antagonist," "anti-IL-31 receptor α antibody," "chronic itch," and "nodular prurigo." In order to find more qualifying publications, we also looked through the reference lists of the included research and pertinent reviews. We also looked through clinical trial databases to find further trials that were pertinent to the subject. The search was performed by two authors. Any disagreement between them was resolved through discussion. 

Study Selection 

All identified records were imported into reference management software (EndNote Version 9; Clarivate, London, UK), and duplicates were removed. Two independent reviewers screened the titles and abstracts to exclude irrelevant studies. Full-text articles were retrieved for potentially eligible studies, and these were assessed against predefined inclusion criteria. We included randomized controlled trials (RCTs) that investigated nemolizumab in patients with prurigo nodularis and reported outcomes of interest. Studies focusing on other conditions, lacking relevant outcomes, or not employing nemolizumab as an intervention were excluded. In addition, we excluded case reports, series, animal studies, and studies lacking a control arm. Discrepancies in study selection were resolved through discussion or consultation with a third reviewer. 

Data Extraction 

A pre-made data extraction form was used to extract the data. Study characteristics (e.g., author, year, design, sample size), patient demographics, treatment plans, and outcomes of interest were among the extracted data. A third reviewer was consulted or discussed with in order to settle disagreements between the reviewers. The following results were evaluated in this study: A decrease of at least four points in the Worst Itch Numeric Rating Scale (WI-NRS) from the beginning to the end of the study is known as the "itch response"; Achievement on the Investigator's Global Assessment (IGA) scale is defined as a score of 0 (clear) or 1 (nearly clear) with a decrease of at least two grades from the baseline; To evaluate safety, all reported treatment-emergent adverse events (AEs) - both serious and non-serious - were retrieved as assessed in individual studies. 

Quality Assessment 

The Cochrane Risk of Bias 2 (RoB 2; Cochrane Collaboration, London, UK) tool was used to evaluate the methodological quality of the included RCTs. The randomisation procedure, departures from planned interventions, missing outcome data, outcome measurement, and choice of reported result are the five areas of bias that are assessed by this method. The evaluations were conducted separately by two reviewers, and conflicts were settled by consensus. 

Data Analysis 

Review Manager (RevMan) Version 5.4.1 (Cochrane Collaboration) was used to synthesise the data. Using random-effects models, we computed pooled estimates of treatment effects to take study heterogeneity into consideration. We provided 95% confidence intervals (CIs) along with risk ratios (RRs) for outcomes. The I2 statistic was used to measure heterogeneity, and thresholds of 25%, 50%, and 75%, respectively, were used to indicate low, moderate, and high heterogeneity. 

Results 

**Figure 1:**
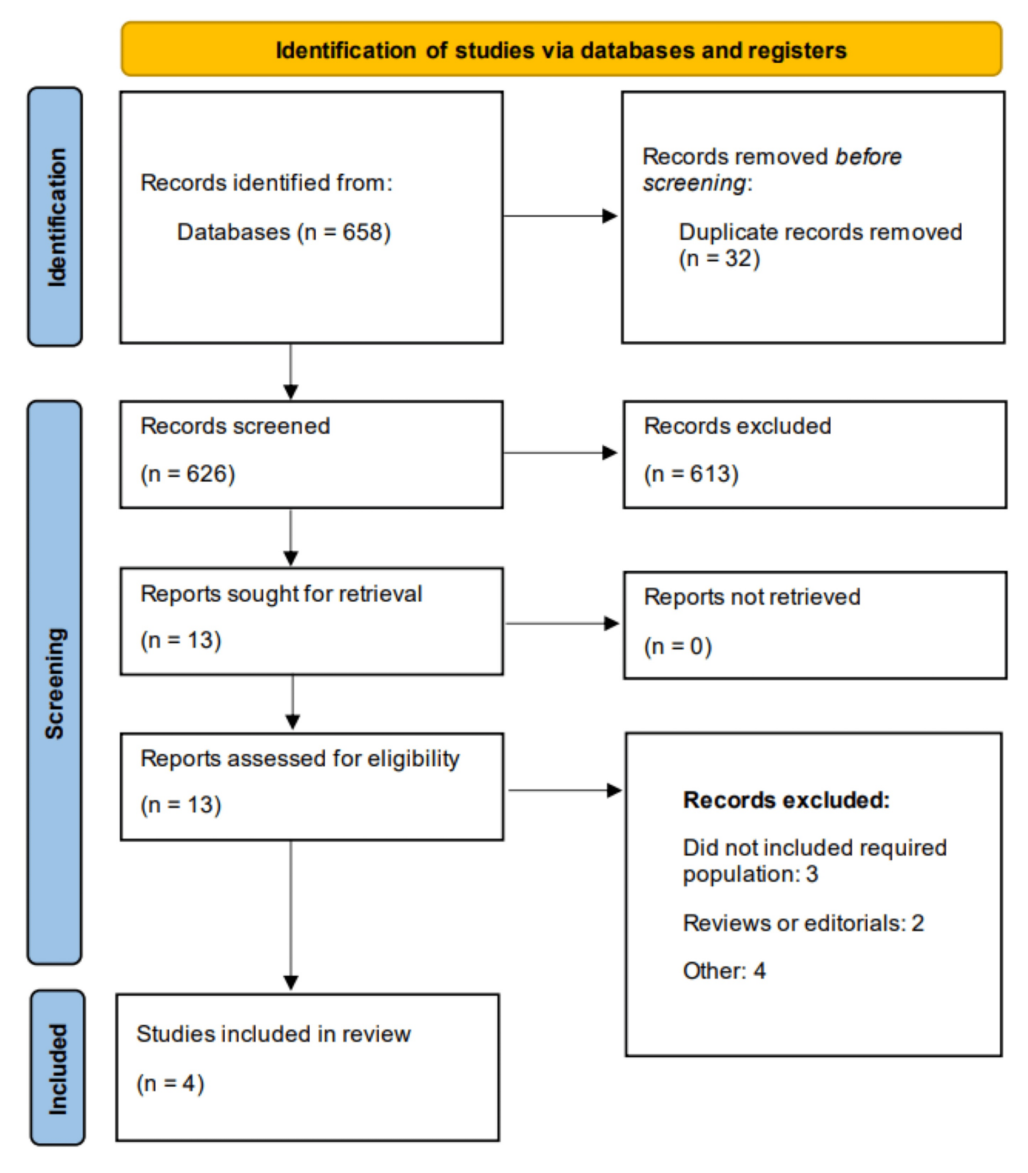
Preferred Reporting of Systematic Review and Meta-Analysis (PRISMA) flowchart- study selection process

The initial search yielded 658 records, of which 32 duplicates were removed. After screening titles and abstracts, 13 studies were deemed potentially eligible and underwent full-text review. A total of four RCTs met the inclusion criteria and were included in the final analysis. The study selection process is illustrated in the PRISMA flow diagram (Figure 1).

**Figure 2:**
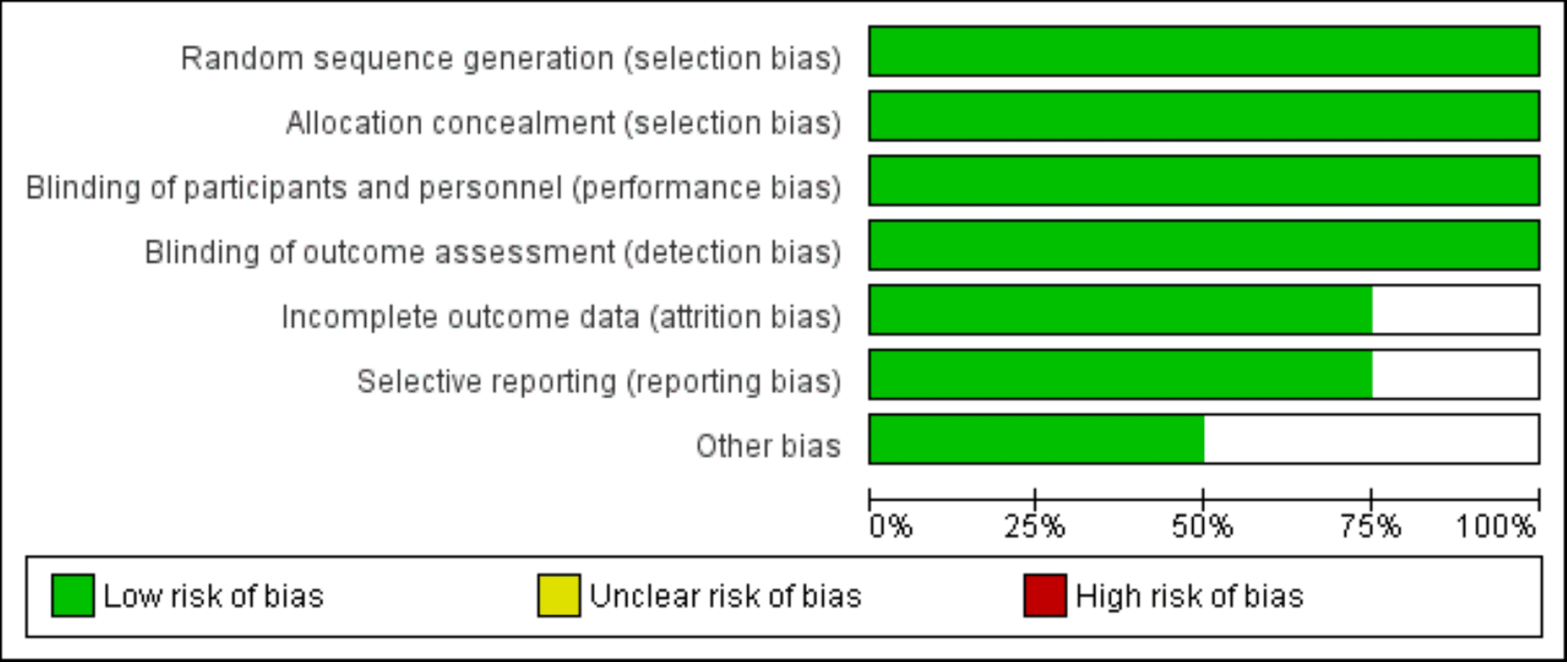
Risk of bias graph of included studies

The included studies were published between 2020 to 2024 and collectively enrolled 859 participants. Sample sizes ranged from 70 to 286 patients per study. All trials investigated the efficacy of nemolizumab in patients with moderate-to-severe prurigo nodularis. The duration of the included studies varied between 11 to 32 weeks. Key characteristics of the studies, including patient demographics and intervention details, are reported in Table 1. Figure 2 shows the risk of bias assessment of included studies.

**Table 1:** Included studies characteristics NR: not reported, RCT: randomized controlled trial

Author	Trial Name	Setting	Groups	Sample Size	Dose of Nemolizumab	Follow-up	Mean Age (Years)	Males (n)
Kwatra et al., 2023 [10]	OLYMPIA 2 Phase 3 RCT	Multicenter	Nemolizumab	183	30 or 60 mg	16 Weeks	53.7	70
			Placebo	91			50.8	36
Stander et al., 2020 [9]	Phase 2 RCT	Multicenter	Nemolizumab	34	0.5 mg per kilogram of body weight	12 Weeks	59.7	15
			Placebo	36			52.4	14
Stander et al., 2024 [11]	OLYMPIA 1 Phase 3 RCT	Multicenter	Nemolizumab	190	30 or 60 mg	32 Weeks	NR	NR
			Placebo	96				
Yokozeki et al., 2024 [12]	Nemolizumab-JP11 Phase 3 RCT	Multicenter	Nemolizumab	153	30 or 60 mg	16 Weeks	50.5	81
			Placebo	76			54	40

Itch Response 

**Figure 3:**
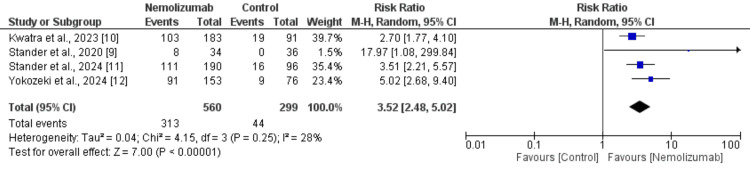
Comparison of itch response between two groups References: [9-12]

Four randomized controlled trials, comprising a total of 859 participants (560 in the nemolizumab group and 299 in the control group), evaluated the effect of nemolizumab on itch response. Pooled analysis demonstrated that nemolizumab significantly improved itch response compared to the control, with an RR of 3.52 (95% CI: 2.48 to 5.02, p < 0.00001) as shown in Figure 3. The heterogeneity among the studies was low (I² = 28%, p = 0.25), indicating consistency in the treatment effect across studies. Individual studies reported RRs ranging from 2.70 to 17.97, all favoring nemolizumab. The results confirm the robust efficacy of nemolizumab in reducing pruritus in patients with prurigo nodularis.

IGA Success 

**Figure 4:**
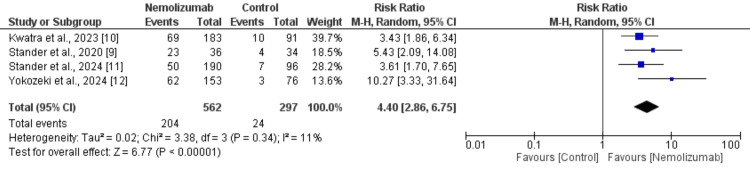
Comparison of Investigator's Global Assessment (IGA) success between two groups References: [9-12]

Four randomized controlled trials, including a total of 859 participants (562 in the nemolizumab group and 297 in the control group), assessed the proportion of patients achieving IGA success. The pooled analysis revealed that nemolizumab significantly increased the likelihood of achieving IGA success compared to the control, with an RR of 4.40 (95% CI: 2.86 to 6.75, p < 0.00001) as shown in Figure 4. Heterogeneity across studies was low (I² = 11%, p = 0.34), indicating consistency in the treatment effect. Individual studies reported RRs ranging from 3.43 to 10.27, all favoring nemolizumab. 

Adverse Events 

**Figure 5:**
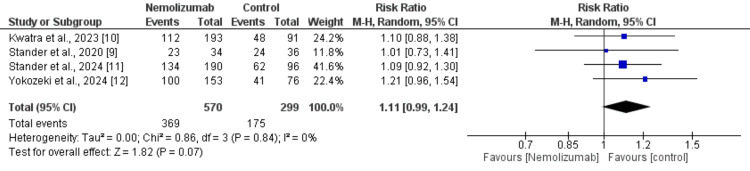
Comparison of adverse events between two groups References: [9-12]

Four studies were used to compare the adverse events between patients who received nemolizumab and patients in the control group and the results are presented in Figure 5. The pooled analysis showed that although the risk of adverse events is higher in the control group but the difference is statistically insignificant (RR: 1.11, 95% CI: 0.99 to 1.24). No heterogeneity was reported among the study results (I² = 0%, p = 0.84). 

Discussion 

This meta-analysis aimed to evaluate the efficacy and safety of nemolizumab in patients with moderate to severe prurigo nodularis. The pooled analysis of four RCTs demonstrated that nemolizumab significantly improved both itch response and IGA success. These findings indicate that nemolizumab effectively enhances overall skin condition in this patient population. Notably, all included trials consistently reported similar results, reinforcing the overall benefits of nemolizumab for managing moderate to severe prurigo nodularis. 

Nemolizumab is a monoclonal antibody that targets the interleukin-31 receptor alpha (IL-31RA), which is key in mediating pruritus and inflammation in skin conditions like prurigo nodularis [13]. IL-31, produced by activated T-helper 2 cells, binds to IL-31RA on sensory neurons and immune cells in the skin, initiating a signaling cascade that causes severe itching and inflammation [14]. By blocking IL-31RA, nemolizumab inhibits this pathway, effectively reducing both the sensation of itching and the associated skin inflammation [15]. This results in improved symptoms in patients with moderate to severe prurigo nodularis, including a reduction in the intensity of itching and the number and severity of prurigo nodules, ultimately improving patient quality of life [12,13]. 

A recent investigation into dupilumab has highlighted the involvement of type 2 inflammation and the interleukin-4-interleukin-13 pathway in the pathophysiology of prurigo nodularis [16]. While the precise molecular and cellular mechanisms of nemolizumab are still under investigation, transcriptomic analysis of skin biopsies from a phase 2 trial in prurigo nodularis patients revealed significant suppression of markers associated with cutaneous nerve activity [17]. Taken together, these findings suggest that targeting the interleukin-31 receptor A provides broad modulation of prurigo nodularis' underlying pathophysiology, further supporting the central role of interleukin-31 in the disease’s progression [16]. 

In this meta-analysis, although the incidence of adverse events was higher in patients receiving nemolizumab, most of the reported events were mild to moderate in severity. Previous studies investigating nemolizumab in patients with atopic dermatitis have noted that peripheral edema and asthma occurred more frequently in the nemolizumab group compared to the placebo group [18,19]. While all the included studies reported atopic dermatitis [9-12], only one study found a higher incidence of atopic dermatitis in the nemolizumab group than in the placebo group. The exact cause of atopic dermatitis in these patients remains not fully understood [10]. The long-term safety and efficacy of nemolizumab are currently being evaluated in the ongoing 192-week OLYMPIA LTE trial. 

Study Limitations 

There are several limitations to this study that should be considered when interpreting the results. Firstly, the analysis included only four RCTs, which may limit the generalizability of the findings. A larger number of trials could provide a more robust and comprehensive assessment of the efficacy and safety of nemolizumab. Secondly, the maximum follow-up duration in the included studies was 32 weeks, which means that long-term efficacy and safety data were not assessed. As a result, the long-term impact of nemolizumab on patients with prurigo nodularis remains unclear, and further studies with extended follow-up periods are necessary to fully understand the drug's enduring effects and potential risks. Thirdly, subgroup analysis was not conducted in this meta-analysis due to a lack of sufficient data. This limitation prevents a more detailed exploration of the treatment’s effects across different patient populations, such as those with varying levels of disease severity or comorbidities. Future trials should aim to address these gaps by including larger, more diverse populations and providing longer-term data to offer a clearer understanding of nemolizumab’s role in the management of prurigo nodularis. 

## Conclusions

The meta-analysis of four RCTs demonstrates that nemolizumab is an effective treatment for moderate to severe prurigo nodularis, showing significant improvements in both itch response (RR: 3.52) and IGA success (RR: 4.40). While adverse events were slightly higher in the treatment group, the difference was not statistically significant. Despite these promising results, the study's limitations, including the small number of trials, relatively short follow-up period, and lack of subgroup analysis, suggest the need for further research. Long-term studies with larger, more diverse patient populations are essential to fully understand nemolizumab's enduring efficacy and safety profile in treating prurigo nodularis.
